# Calibrated Transformer Fusion for Dual-View Low-Energy CESM Classification

**DOI:** 10.3390/jimaging12010041

**Published:** 2026-01-13

**Authors:** Ahmed A. H. Alkurdi, Amira Bibo Sallow

**Affiliations:** 1 Department of Information Technology, Technical College of Duhok, Duhok Polytechnic University, Duhok 42001, Iraq; amira.bibo@dpu.edu.krd; 2 Department of Information Technology, Technical College of Informatics-Akre, Akre University for Applied Sciences, Aqrah 42003, Iraq

**Keywords:** contrast-enhanced spectral mammography (CESM), deep learning, transformer, dual-view fusion, breast abnormality detection, uncertainty estimation, medical image analysis

## Abstract

Contrast-enhanced spectral mammography (CESM) provides low-energy images acquired in standard craniocaudal (CC) and mediolateral oblique (MLO) views, and clinical interpretation relies on integrating both views. This study proposes a dual-view classification framework that combines deep CNN feature extraction with transformer-based fusion for breast-side classification using low-energy (DM) images from CESM acquisitions (Normal vs. Tumorous; benign and malignant merged). The evaluation was conducted using 5-fold stratified group cross-validation with patient-level grouping to prevent leakage across folds. The final configuration (Model E) integrates dual-backbone feature extraction, transformer fusion, MC-dropout inference for uncertainty estimation, and post hoc logistic calibration. Across the five held-out test folds, Model E achieved a mean accuracy of 96.88% ± 2.39% and a mean F1-score of 97.68% ± 1.66%. The mean ROC-AUC and PR-AUC were 0.9915 ± 0.0098 and 0.9968 ± 0.0029, respectively. Probability quality was supported by a mean Brier score of 0.0236 ± 0.0145 and a mean expected calibration error (ECE) of 0.0334 ± 0.0171. An ablation study (Models A–E) was also reported to quantify the incremental contribution of dual-view input, transformer fusion, and uncertainty calibration. Within the limits of this retrospective single-center setting, these results suggest that dual-view transformer fusion can provide strong discrimination while also producing calibrated probabilities and uncertainty outputs that are relevant for decision support.

## 1. Introduction

Breast cancer is now the most commonly diagnosed cancer globally, representing 11.7% of all new cancer cases (1 in 4 among women) and 6.9% of all cancer deaths (1 in 6 among women) worldwide [1]. Early detection through imaging significantly improves survival rates, with mammography continuing to serve as the clinical gold standard for screening and diagnosis. Traditional full-field digital mammography (FFDM) offers high spatial resolution but limited sensitivity in dense breast tissues, prompting the development of contrast-enhanced spectral mammography (CESM) as an advanced alternative. CESM enhances lesion conspicuity by combining low- and high-energy acquisitions, improving the differentiation of benign and malignant abnormalities [2].

Deep learning (DL) has revolutionized computer-aided diagnosis in mammography, enabling automated feature extraction and classification with performance approaching expert radiologists [3]. Convolutional neural networks (CNNs) such as ResNet and DenseNet have been successfully applied to mammogram analysis for lesion detection, segmentation, and malignancy prediction [4]. However, most CNN-based methods are limited to single-view processing or rely on simplistic feature concatenation, which cannot capture cross-view dependencies beyond simple aggregation between the two standard mammographic views—craniocaudal (CC) and mediolateral oblique (MLO). In contrast, radiologists interpret these views jointly to exploit their complementary anatomical information, suggesting that dual-view integration is critical for robust automated diagnosis [5].

Recent innovations using Vision Transformers (ViTs) have introduced self-attention mechanisms capable of modeling long-range dependencies across multiple views or modalities. Hybrid CNN–Transformer architectures have achieved state-of-the-art accuracy in mammographic classification by combining local texture encoding with global contextual reasoning [6,7]. Furthermore, models such as NaFV-Net [8] and Mam-Incept-Net [9] demonstrated that attention-based fusion of multiple mammographic views substantially improves lesion localization and reduces false positives compared to CNN-only pipelines.

In the context of contrast-enhanced spectral mammography (CESM), multi-view and multi-phase data introduce both opportunities and practical challenges for automated analysis. The CDD-CESM dataset [10] provides a useful benchmark for developing and evaluating such architectures. However, dual-view Transformer-based fusion in CESM remains underexplored, particularly when probability outputs are intended for decision support and therefore require uncertainty and calibration reporting [7,11].

To address this gap, the present study introduces Dual View Transformed Fusion, which formulates dual-view fusion at the token level and models complementary dependencies across views and feature extractors. Each breast side is represented using two ImageNet-pretrained CNN backbones (ResNet-101 and DenseNet-121) applied to both the craniocaudal (CC) and mediolateral oblique (MLO) projections, yielding four view-specific embeddings. These embeddings are mapped into a shared representation space and fused using a lightweight TransformerEncoder operating on a short token sequence augmented with a learnable classification token.

Beyond discrimination metrics, the framework also reports decision-support outputs by integrating Monte Carlo dropout for uncertainty estimation with per-fold post hoc logistic calibration. Probability quality is quantified using the Brier score, expected calibration error, and negative log-likelihood under patient-disjoint cross-validation. Accordingly, the contribution is not limited to dual-view fusion performance, but also includes an uncertainty-aware and calibrated probability pipeline evaluated for side-level triage on CDD-CESM.

## 2. Related Work

Deep learning (DL) has become the cornerstone of automated mammographic analysis due to its ability to learn discriminative visual representations directly from imaging data. Convolutional neural networks (CNNs) such as ResNet, DenseNet, and Inception architectures have shown remarkable accuracy in classifying breast lesions and segmenting regions of interest (ROIs) from mammograms [3]. However, CNNs are inherently limited in modeling global contextual dependencies, particularly when applied to multi-view or multi-phase modalities such as contrast-enhanced spectral mammography (CESM).

To address this, several studies introduced attention mechanisms and Vision Transformers (ViTs) into mammographic pipelines, significantly improving classification performance. Ref. [6] proposed a hybrid CNN–ViT model that combined convolutional feature maps with transformer encodings, achieving a substantial improvement in sensitivity for microcalcification classification. Similarly, ref. [7] developed an attention-based multi-scale fusion transformer that effectively captured inter-view correlations in dual-view mammograms, outperforming single-view baselines on the INbreast and CBIS-DDSM datasets.

Traditional radiological interpretation relies on dual-view analysis—the craniocaudal (CC) and mediolateral oblique (MLO) views—to provide complementary anatomical context. Early computer-aided systems, however, typically processed these views independently. Ref. [12] introduced a dual-view mass matching framework for breast cancer detection that used cross-view correspondence learning to improve mass localization accuracy. Building on this concept, ref. [8] proposed NaFV-Net, an adversarial four-view architecture integrating CNN backbones and self-attention mechanisms for enhanced lesion classification.

Ref. [9] later extended this idea through Mam-Incept-Net, which incorporated inception modules and attention-driven feature aggregation, achieving improved interpretability and higher AUC scores. More recently, ref. [13] developed a multi-view co-occurrence and dual-modality fusion framework that integrates mammography and ultrasound data, underscoring the growing role of multimodal fusion in clinical AI for breast cancer diagnosis.

Contrast-Enhanced Spectral Mammography (CESM) has emerged as a powerful modality, providing both low-energy (LE) and recombined (subtracted) images that enhance lesion conspicuity. Deep learning models tailored to CESM data have demonstrated strong potential in differentiating malignant from benign lesions. Ref. [14] presented a multiprocess DL system integrating multi-view CESM images with auxiliary fusion layers, showing significant diagnostic improvements in a multicenter study. Prior CESM-related deep learning work has also leveraged CC and MLO views jointly, including concatenation-based dual-view fusion, which supports treating feature concatenation as a baseline for multi-view integration in this study [15].

Ref. [4] proposed DualNet, a two-stream CNN architecture specifically designed for CESM-based classification, using improved watershed segmentation to enhance ROI delineation. In parallel, ref. [11] demonstrated that ROI-stratified CNN–Transformer hybrids can effectively leverage CESM contrast information for malignancy discrimination.

Despite these advances, most CESM-focused models still process views or contrast phases separately. There remains a need for architectures that jointly model dual-view representations, capturing both spatial and contextual interdependencies.

The recent literature has underscored the transformative potential of Vision Transformers in multi-view medical imaging. Ref. [16] proposed a multi-scale Swin Transformer framework capable of integrating features from multiple mammographic views and contrast levels, showing superior interpretability. Ref. [7] further advanced this direction with a hybrid-view transformer fusion strategy combining CNN-extracted features and self-attention-based cross-view alignment.

Comprehensive reviews [2,3,5] affirm that hybrid CNN–Transformer designs will continue to define the next generation of intelligent mammography systems, offering more transparent, data-efficient, and clinically reliable solutions. However, integrating dual-view fusion within CESM remains an underexplored yet promising research avenue—one that this study directly addresses.

## 3. Materials and Methods

This study is reported in accordance with the Checklist for Artificial Intelligence in Medical Imaging (CLAIM) and the TRIPOD + AI statement for prediction model reporting [17,18]. The experiments were conducted using the CDD-CESM dataset [10] and formulated as side-level binary classification (Normal vs. Tumorous), where benign and malignant findings were merged into a single tumorous class. Model development and evaluation followed a patient-grouped 5-fold stratified cross-validation protocol to prevent leakage across folds.

### 3.1. Dataset and Task Definition

The experiments were conducted using the Categorized CESM dataset (CDD-CESM) [10]. The CDD-CESM dataset contains 326 patients and 2006 images, including 1003 low-energy (DM) images and 1003 recombined images. Images are provided for left and right breasts in CC and MLO views, although some patients have missing views and a small number have duplicate acquisitions. In this study, only low-energy images were used. Side-level samples were formed at the breast-side level, resulting in 566 breast-side inputs across 326 patients. Of these, 435 sides contain both CC and MLO views, while 131 sides contain a single available view (65 CC-only and 66 MLO-only). For single-view sides, the missing view was handled by zero-padding to maintain a fixed two-view tensor representation. Duplicate acquisitions were identified when more than one low-energy image existed for the same patient, breast side, and view (CC or MLO). To avoid disproportionate weighting of individual patients, a single acquisition was retained per patient–side–view, and all remaining duplicates were excluded before tensor construction and fold generation. The retained acquisition was selected deterministically (first occurrence in the dataset ordering) to ensure reproducibility.

At the label level, CDD-CESM provides Normal, Benign, and Malignant annotations. For the binary task, Benign and Malignant were merged into a single Tumorous class to support dual-view triage under limited sample size. This endpoint corresponds to an abnormality triage setting, where the primary decision is whether a breast side contains any suspicious finding that warrants further assessment. Accordingly, benign findings were treated as positive together with malignant findings, while benign–malignant discrimination was not targeted in this study. The final binary distribution used in the experiments was Normal = 189 and Tumorous = 377. The tri-class distribution was Normal = 189, Benign = 194, and Malignant = 183.

Side-level labels were derived using a worst-case aggregation rule across the available views for the same breast side. Specifically, if CC and MLO labels differed, the side label was set to the higher-severity class (Malignant > Benign > Normal). Table S1 summarizes the dataset composition and the derived side-level sample counts.

### 3.2. Input Construction and Preprocessing

**Figure 1:**
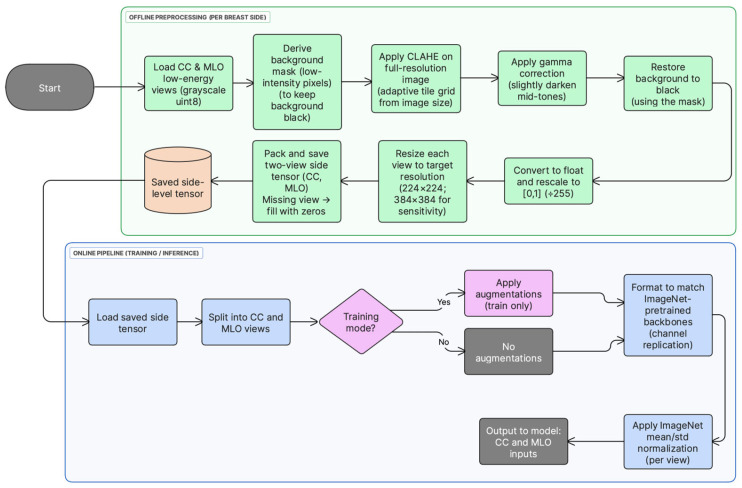
Preprocessing and input construction pipeline for low-energy CC and MLO views. Offline: full-resolution CLAHE-based enhancement, gamma correction, background masking, scaling to [0, 1], resizing, and tensor packing. Online (load time): grayscale-to-pseudo-RGB conversion, optional augmentation (training only), and ImageNet normalization.

All low-energy CESM images were preprocessed offline before model training. First, each CC/MLO view was loaded as an 8-bit grayscale image. Second, a background mask was derived from the original image (pixel values ≤ 5) and used to keep the background black after enhancement. Third, local contrast was enhanced on the full-resolution image using contrast-limited adaptive histogram equalization (CLAHE) with clipLimit = 1.0 and an adaptive tileGridSize selected from the image size to approximate 1000-pixel tiles (grid = (round (w/1000), round (h/1000)), clipped to [4, 64] tiles per axis). Fourth, gamma correction (γ = 2.5) was applied to slightly darken mid-tones while preserving bright structures, and background pixels were restored to zero using the mask. Fifth, the enhanced image was converted to float and rescaled to [0, 1] by dividing by 255, and then resized to 224 × 224 pixels. Finally, for each breast side, the processed CC and MLO views were stored as two grayscale channels in a tensor; if a view was unavailable, its channel was filled with zeros. This offline pipeline enforced identical preprocessing across folds and reduced runtime overhead. Preprocessing choices follow established mammography deep learning pipelines [7,12]. Figure 1 summarizes the preprocessing and input construction steps used in this study.

During training, lightweight data augmentation (random horizontal flips and small rotations) was applied at load time. Each grayscale view was then converted to a 3-channel pseudo-RGB input by channel replication to enable direct use of ImageNet-pretrained CNN backbones, followed by standard ImageNet mean/std normalization. In contrast, CLAHE-based enhancement (including gamma correction and background masking) and tensor construction were performed offline and remained fixed across folds.

If either CC or MLO was unavailable for a breast side, the corresponding missing view channel was filled with an all-zero image (224 × 224 or 384 × 384), so the network always received a fixed two-view tensor; after ImageNet normalization this missing input becomes a constant-valued map.

To assess sensitivity to input resolution, an additional tensor set was generated directly from the original low-energy images at 384 × 384 resolution (i.e., not upsampled from 224 × 224 tensors). The same offline preprocessing pipeline was applied at native resolution, including full-resolution CLAHE-based enhancement, gamma correction, background masking, and rescaling to ([0, 1]), followed by resizing to 384 × 384 and two-view tensor packing. The same training and evaluation protocol was then used. As in the main setting, pseudo-RGB conversion, optional augmentation (training only), and ImageNet normalization were applied at load time.

### 3.3. Data Augmentation

Light augmentation was applied during training to reduce overfitting while preserving mammographic structure. Random horizontal flipping with probability 0.5 and random rotation within ±10° were applied. Validation and test samples were processed without augmentation. This ensured that reported test performance reflected generalization under a consistent input distribution.

Augmentations were applied consistently at the breast-side level. For each side-level sample, the flip decision and rotation angle were sampled once and then applied identically to both CC and MLO images to preserve interprojection geometric consistency required for fusion learning. Therefore, CC/MLO alignment was not artificially disturbed by independent view-wise augmentation. Validation and test samples were processed without augmentation.

### 3.4. Cross-Validation Protocol and Leakage Control

A 5-fold StratifiedGroupKFold protocol was used. Stratification was performed on the binary label, and grouping was performed by patient ID. This ensured that samples from the same patient were not shared across training and test folds. Since radiological views from the same patient are correlated, this constraint was applied to prevent leakage and optimistic performance estimates.

Within each training fold, a validation subset was created by holding out 15% of patients using patient-level stratification, so validation remained patient-disjoint from both training and the held-out test fold. The resulting subsets were used as follows:

- The training subset was used for optimization,
- The validation subset was used for early stopping and threshold selection, and
- The held-out fold was used only for final testing.

To prevent leakage, all samples were grouped by patient identifier, such that both views (CC/MLO) and both breast sides from the same patient were always assigned to the same fold. After fold generation, split integrity was verified by checking that the intersection of patient IDs between training, validation, and test partitions was empty for every fold (i.e., zero shared patients across partitions).

### 3.5. Class Imbalance Handling

Class imbalance was addressed in two steps. First, a WeightedRandomSampler was applied to the training subset using inverse class frequency. Second, class-weighted cross-entropy loss was used, where weights were computed from the training fold distribution. This combination was applied to stabilize learning and to reduce bias toward the majority class.

### 3.6. Dual-Backbone Feature Extraction

Dual-view representation learning was performed using two ImageNet-pretrained CNN backbones, ResNet-101 and DenseNet-121. For ResNet-101, the network was truncated before the original pooling and classification layers, and the final convolutional feature map was aggregated using global average pooling to obtain a 2048-dimensional vector. This vector was then mapped to a 1024-dimensional embedding using a learnable linear projection (2048 → 1024). For DenseNet-121, the final convolutional feature map was likewise globally pooled to obtain a 1024-dimensional vector, followed by a linear mapping to 1024 dimensions (1024 → 1024). A 1024-dimensional embedding size was adopted to match the transformer fusion dimension while keeping fusion complexity moderate. Finally, embeddings were L2-normalized prior to fusion to stabilize token scale.

**Figure 2:**
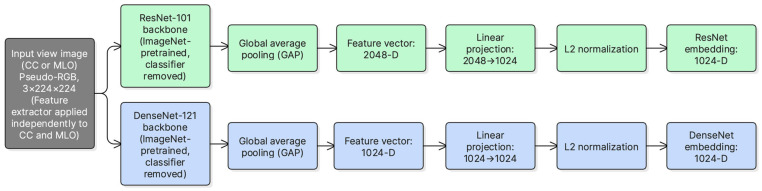
ResNet-101 and DenseNet-121 feature extraction modules used to obtain view-level embeddings from CC and MLO inputs.

Both CC and MLO views were passed through each backbone, producing four embeddings per breast side. This design enables complementary feature extraction, where global structures and fine-grained texture patterns can be captured through different backbone characteristics. Figure 2 illustrates the feature extraction modules used for ResNet-101 and DenseNet-121.

For each breast side, two low-energy views are available (CC and MLO). Each view is encoded using two CNN backbones, ResNet-101 and DenseNet-121. This produces four embeddings:

$$F_{CC}^{\left(r\right)},\;F_{MLO}^{\left(r\right)},\;F_{CC}^{\left(d\right)},\;F_{MLO}^{\left(d\right)},$$

where r and d denote ResNet and DenseNet, respectively. Each embedding is projected into a common D-dimensional space (here D=1024) and L2-normalized to keep token scales consistent across backbones.

A learnable classification token $[\mathrm{CLS}]\in R^{D}$ is prepended to form a token sequence of length 5:

(1) $$X=[x_{\mathrm{CLS}};\;F_{CC}^{\left(r\right)};\;F_{MLO}^{\left(r\right)};\;F_{CC}^{\left(d\right)};\;F_{MLO}^{\left(d\right)}]\in R^{5\times D}.$$

This construction preserves view identity and backbone diversity before fusion.

### 3.7. Transformer-Based Cross-View Fusion (DVTF)

After feature extraction, the four view-backbone embeddings were projected into a unified 1024-dimensional space and L2-normalized. A learnable [CLS] token was prepended to form a sequence of five tokens. This sequence was passed to a 2-layer TransformerEncoder to model cross-view and cross-backbone dependencies through multi-head self-attention and feed-forward sublayers. In this implementation, the transformer encoder consisted of two encoder layers with 4 attention heads (d = 1024) and a 4096-dimensional feed-forward sublayer (i.e., 4 × d). GELU was employed as the activation function, and dropout (*p* = 0.2) was applied within the attention and feed-forward sublayers. In addition, a learnable positional embedding was added to the 5-token sequence ([CLS] + four view tokens) prior to fusion. This approach aligns with prior work that uses attention mechanisms to model inter-view relationships beyond simple feature concatenation [6,8].

**Figure 3:**
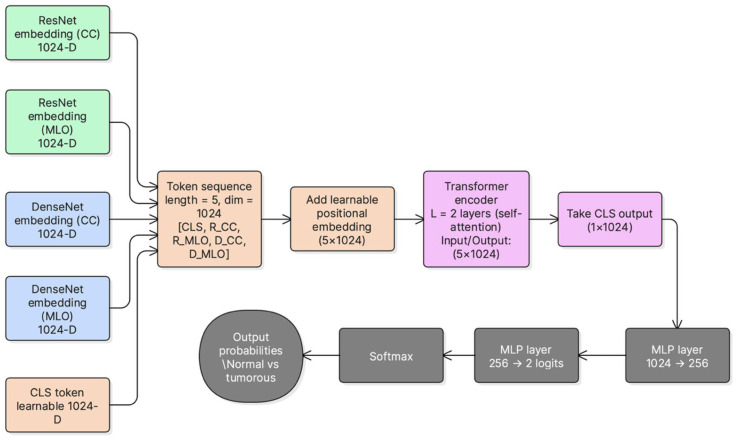
DVTF architecture for dual-view fusion. CC and MLO are encoded by ResNet-101 and DenseNet-121, projected into a unified embedding space, fused using a TransformerEncoder with a learnable [CLS] token, and classified using an MLP head.

The final [CLS] embedding was used as the fused representation for classification. A two-layer MLP head (1024 → 256 → 2) produced logits for Normal vs. Tumorous classification. Figure 3 summarizes the fusion architecture used in DVTF.

The token sequence X is processed using a TransformerEncoder to model cross-view and cross-backbone dependencies through self-attention [6,8]. For each encoder layer, the update can be written as:

(2) Z=MSA(X)+X,

(3) H=FFN(Z)+Z,

where MSA denotes multi-head self-attention and FFN denotes the position-wise feed-forward network. In practice, attention is implemented through learned query, key, and value projections ($W_{Q},W_{K},W_{V}$) as in standard transformer formulations [6,8]. The final fused representation is taken from the output $\left[\mathrm{CLS}\right]$ token. This fused feature is then passed to the MLP classifier to predict Normal versus Tumorous.

Attention-based refinement mechanisms have been used in medical image analysis to combine global contextual cues with local detail and to focus feature integration on anatomically relevant regions. This precedent supports using self-attention to promote cross-view feature interaction beyond simple aggregation [19].

### 3.8. Uncertainty Estimation Using MC-Dropout

**Figure 4:**
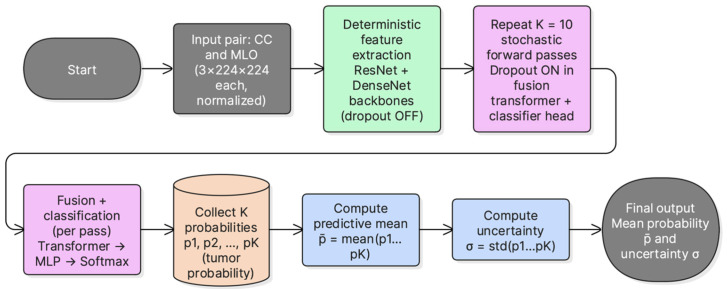
MC-dropout inference pipeline. Multiple stochastic forward passes are performed with active dropout, the mean tumorous probability is used for prediction, and the standard deviation is used as an uncertainty score.

Epistemic uncertainty was estimated using Monte Carlo dropout during inference. At test time, dropout layers were kept active and multiple stochastic forward passes were performed per sample. The mean tumorous probability across passes was used as the predictive probability. The standard deviation of the tumorous probability across passes was used as an uncertainty score. This provides a simple uncertainty estimate without modifying the base architecture. Figure 4 summarizes the inference procedure used to obtain the MC mean probability and uncertainty score.

MC Dropout was performed with K = 10 stochastic forward passes per sample. Dropout (*p* = 0.2) was enabled during inference for the transformer encoder and classification head. No dropout layers are present in the ResNet-101 and DenseNet-121 feature extractors; therefore, the stochasticity introduced by MC-Dropout is confined to the fusion encoder and classification head. The predictive probability was computed as the mean of the K softmax outputs, and uncertainty was quantified as the standard deviation of the Tumorous-class probability. A logistic calibration model was then fitted per fold on the validation MC probabilities and applied to the corresponding test fold.

### 3.9. Post Hoc Logistic Calibration

Post hoc logistic calibration was applied to improve probability behavior. For each fold, calibration parameters were learned using the validation subset. The calibrator was applied to the MC mean probability using a logistic mapping in logit space:

(4) $$\mathrm{logit}(p_{cal})=a\cdot \mathrm{logit}(p_{mc})+b$$

The learned parameters were then applied to the test MC mean probabilities to obtain calibrated probabilities. This step transforms confidence values via a 1D logistic mapping and typically preserves ranking when the fitted mapping is monotonic.

### 3.10. MC-Dropout K-Sensitivity and Runtime

The K-sensitivity analysis was conducted as an independent rerun of the full training and evaluation pipeline under the same 5-fold protocol. In the primary uncertainty experiments, the number of MC-dropout stochastic forward passes was set to K=10. In addition, a sensitivity analysis was performed using K∈{5,10,20} to quantify the effect of K on discrimination, calibration, and runtime. For each fold and each K, MC mean probabilities were computed on the validation subset, and a fold-specific logistic calibrator was fitted using the corresponding validation labels. The fitted calibrator was then applied to the test MC mean probabilities prior to metric computation. Latency was measured on an NVIDIA A100 GPU using batch size 16 and is reported as milliseconds per sample and per batch. This analysis provides a practical trade-off between calibration quality and computational cost.

### 3.11. Ablation Study Design (Models A–E)

An ablation study was conducted to quantify the incremental contribution of each design component. All models used the same dataset splits, transforms, optimization schedule, and evaluation pipeline. The evaluated configurations were:

- Model A: simple CNN baseline using LE CC without transformer fusion and without MC-dropout;
- Model B: dual-view CNN baseline using conventional feature fusion without transformer fusion and without MC-dropout;
- Model C: deterministic DVTF using dual backbones and transformer fusion without MC-dropout;
- Model D: DVTF with MC-dropout inference, reporting MC mean probability and uncertainty;
- Model E: DVTF with MC-dropout inference and post hoc logistic calibration. Model E was treated as the main model in this paper.
- Fusion baseline (Weighted): learnable weighted averaging over the four tokens ResNet(CC),ResNet(MLO),DenseNet(CC),DenseNet(MLO), followed by an MLP head (evaluated with MC-dropout + logistic calibration);
- Fusion baseline (Gated): feature-wise gating between CC and MLO per backbone, then backbone fusion by concatenation + projection (evaluated with MC-dropout + logistic calibration);
- Fusion baseline (Cross-attention): two-token attention between CC,MLO per backbone, then backbone fusion by concatenation + projection (evaluated with MC-dropout + logistic calibration).

The three fusion baselines were added to isolate the contribution of the DVTF transformer fusion beyond simple token weighting, view-gating, and two-token cross-attention. All baselines retained the same feature extractors and the same outer 5-fold patient-disjoint protocol, and they were evaluated using the same MC-dropout inference (K = 10) and per-fold logistic calibration procedure used for Model E, to ensure a like-for-like comparison of probabilistic outputs.

*Model A (single-view CNN baseline):* Model A used a single low-energy CC view for each breast side. The input was obtained from the pre-created tensor representation by selecting channel 0 (CC LE), while the MLO channel was ignored. A DenseNet-121 backbone pre-trained on ImageNet was employed as a feature extractor. The final convolutional features were passed through ReLU and aggregated using global average pooling, producing a 1024-dimensional feature vector (DenseNet-121 classifier input). Classification was then performed using a lightweight MLP head with the structure 1024 → 256 → 2, using ReLU and dropout (*p* = 0.3). This baseline is deterministic and does not use attention or transformer-based fusion.

(5) $$f_{CC}=g(x_{CC}),\;\hat{y}=MLP(f_{CC})$$

where g(·) is DenseNet-121 with global average pooling.

*Model B (dual-view CNN baseline):* Model B employed a shared DenseNet-121 backbone (ImageNet-pretrained) to extract features independently from the CC and MLO low-energy views. For each view, the final convolutional feature map was passed through ReLU and aggregated using global average pooling, yielding a 1024-dimensional vector. The two view vectors were then combined using feature concatenation to form a 2048-dimensional fused representation. Finally, classification was performed using a lightweight MLP head (2048 → 256 → 2) with ReLU and dropout (*p* = 0.3). No transformer or attention module was used, and fusion was implemented as deterministic late fusion by concatenation.

(6) $$f_{CC}=g(x_{CC}),\;f_{MLO}=g(x_{MLO}),\;z=[f_{CC};f_{MLO}]\;,\hat{y}=MLP(z).$$

*Model C (DVTF without MC-dropout):* Model C implemented dual-view transformer fusion at the breast-side level. For each tensor, the CC low-energy image (channel 0) and MLO low-energy image (channel 1) were extracted. Each view was converted to pseudo-RGB and processed using the same ImageNet normalization used across models. Two ImageNet-pretrained backbones were employed in parallel: ResNet-101 and DenseNet-121. From each backbone, a 1024-dimensional L2-normalized embedding was produced for each view using global average pooling followed by a linear projection. This produced four tokens per side: $\left[\mathrm{CC}\to \mathrm{ResNet},\;\mathrm{MLO}\to \mathrm{ResNet},\;\mathrm{CC}\to \mathrm{DenseNet},\;\mathrm{MLO}\to \mathrm{DenseNet}\right]$. A learnable [CLS] token and learned positional embeddings were appended to form a sequence of 5 tokens of dimension 1024. The token sequence was then fused using a 2-layer Transformer encoder with 4 attention heads and a feed-forward dimension of 4×1024, using GELU activations and dropout p=0.2. The final [CLS] embedding was mapped to logits using a lightweight classifier head 1024 → 256 → 2 with GELU and dropout p=0.2. Model C is evaluated deterministically using a single forward pass at test time; thus, dropout is not used for Monte Carlo sampling and no calibration model is fitted.

(7) $$z=\mathrm{Transformer}([\mathrm{CLS},t_{1},\ldots ,t_{4}]);\;\hat{y}=\mathrm{MLP}(z_{\mathrm{CLS}})$$

where $t_{i}\in R^{1024}$ are view–backbone tokens.

*Model D (DVTF with MC-dropout):* Model D used the same dual-view transformer fusion architecture as Model C, but enabled Monte Carlo (MC) dropout during inference to estimate predictive uncertainty. The CC and MLO low-energy images were extracted from tensor channels 0 and 1, converted to pseudo-RGB, and normalized using ImageNet statistics. Two ImageNet-pretrained feature extractors (ResNet-101 and DenseNet-121) generated four 1024-D L2-normalized tokens per side (CC/MLO × backbone). A learnable [CLS] token and learned positional embeddings formed a 5-token sequence that was fused by a 2-layer Transformer encoder (4 attention heads, GELU activations, dropout *p* = 0.2). The resulting [CLS] embedding was classified using a 1024 → 256 → 2 head with dropout. During training, the model used deterministic dropout as regularization. During evaluation, dropout layers were explicitly kept in training mode and K = 10 stochastic forward passes were performed per sample. The final probability was computed as the mean softmax probability across the K samples, and uncertainty was defined as the standard deviation of the Tumorous-class probability across the K samples. All discrimination and calibration metrics (ROC-AUC, PR-AUC, Brier score, and ECE) were computed from the MC mean probability.

(8) $$\hat{p}=\frac{1}{K}\sum_{k=1}^{K}p^{\left(k\right)},\;\mathrm{and}\;u=\mathrm{std}({\left\{p_{\mathrm{tum}}^{\left(k\right)}\right\}}_{k=1}^{K}),\;\mathrm{with}\;K=10$$

*Model E (DVTF with MC-dropout and logistic calibration):* Model E extended Model D by applying a per-fold post hoc logistic calibration to the MC-dropout probabilities. The same DVTF backbone and fusion procedure were used, with CC and MLO low-energy images taken from tensor channels 0 and 1, converted to pseudo-RGB, and normalized using ImageNet statistics. For each side, four 1024-D L2-normalized tokens (CC/MLO × ResNet-101/DenseNet-121) were fused using a 2-layer Transformer encoder (4 heads, GELU, dropout p=0.2) with a learnable [CLS] token and learned positional encodings. At inference, dropout layers were kept active and *K* = 10 stochastic forward passes were performed per sample. The MC mean Tumorous probability $\hat{p}$ was then calibrated using a validation-fitted 1D logistic mapping of the form $logit(p_{cal})=alogit(\hat{p})+b$. The calibration parameters $\left(a,b\right)$ were learned on the validation set of each fold by minimizing binary cross-entropy for 500 optimization steps (Adam, learning rate 0.01). The fitted mapping was then applied to the corresponding fold test set to obtain calibrated probabilities $p_{cal}$. Threshold selection for F1 was performed on the calibrated validation probabilities, and all reported discrimination and calibration metrics (ROC-AUC, PR-AUC, Brier score, and ECE) were computed using the calibrated test probabilities. Uncertainty was retained from the MC-dropout procedure and reported as the standard deviation of the Tumorous probability across the K samples (identical to Model D).

This design enables direct comparison of conventional dual-view fusion versus transformer-based fusion, and deterministic inference versus uncertainty-aware and calibrated inference. In addition, component-wise ablation is reported to quantify the contribution of each architectural block, following common practice in medical imaging method development where module-level gains are explicitly isolated [19].

### 3.12. Evaluation Metrics and Threshold Selection

Performance was evaluated using accuracy, sensitivity, specificity, precision, and F1-score, as well as ROC-AUC and PR-AUC. Probability quality was evaluated using the Brier score and Expected Calibration Error (ECE) computed using 10 equal-width bins. Negative log-likelihood (NLL) was additionally reported as a proper scoring rule, computed from the calibrated tumorous probability p as $-\frac{1}{N}\sum_{i=1}^{N}[y_{i}log(p_{i})+(1-y_{i})log(1-p_{i})]$, where lower values indicate better probabilistic calibration.

For each fold, Monte Carlo dropout was first applied on the validation subset to obtain mean tumorous probabilities. Next, a logistic calibrator was fitted on the validation outputs and applied to the same validation probabilities. The fold-specific operating threshold $\tau_{f}$ was then selected by sweeping τ∈{0.05,0.10,…,0.95} and maximizing validation F1 on the calibrated validation probabilities $p_{val,cal}$. Finally, the same calibrator was applied to the test probabilities and the selected $\tau_{f}$ was applied to $p_{test,cal}$ to obtain discrete predictions.

After post hoc calibration, predicted probabilities were converted into discrete class labels using the same decision threshold employed for reporting accuracy, sensitivity, specificity, precision, and F1-score. Confusion matrix counts were then computed with Normal treated as the negative class and Tumorous treated as the positive class. To match the pooled ROC/PR analysis, counts were aggregated across the five held-out folds using out-of-fold test predictions.

In each fold, splitting was performed at the patient level using the patient identifier as the grouping variable. Thus, all samples from the same patient (including both CC and MLO views, and both breast sides if present) were assigned to the same split. Within each training fold, the validation subset was created by holding out 15% of patients (not samples), and therefore remained patient-disjoint from both training and test data.

In addition, sensitivity at fixed specificity (0.90, 0.95, and 0.97) was evaluated by selecting a decision threshold on the validation subset of each fold to achieve at least the target specificity, and then reporting the resulting test sensitivity and specificity at that threshold.

This protocol enables patient-grouped evaluation, supports consistent model comparison in the ablation, and reports both discrimination and probability quality.

### 3.13. Training and Implementation Settings

Experiments were conducted in Google Colab using an NVIDIA A100-SXM4-40GB GPU (40 GB). The environment used Python 3.12.12 and PyTorch 2.9.0 + cu126 (CUDA 12.6, cuDNN 91002). Optimization used Adam with a learning rate of 1 × 10^−4^ and weight decay of 1 × 10^−4^, with cosine annealing (T_max = 30, η_min = 1 × 10^−6^). Training used batch size 16 and a maximum of 60 epochs, with early stopping based on validation ROC-AUC (patience = 10). MC-dropout used K = 10 passes and logistic calibration was fitted per fold using the validation subset. Full settings are summarized in Table S2.

### 3.14. Fusion Token Attention and Grad-CAM Qualitative Explanation

To provide qualitative evidence of how the proposed dual-view fusion operates, two complementary visualizations were generated. First, token-level attention weights were extracted from the fusion transformer to illustrate interactions between the five tokens [CLS],Res−CC,Res−MLO,Den−CC,Den−MLO. Attention was taken from the second (final) transformer encoder layer and averaged across heads, then displayed as a 5×5 token-to-token matrix (rows as query tokens and columns as key tokens). This visualization reflects cross-token weighting at the embedding level rather than pixel-level saliency. Second, Grad-CAM was applied to the ResNet stream to localize image regions contributing to the decision in each view. Grad-CAM heatmaps were computed using the final convolutional block and overlaid on the low-energy CC and MLO images. The attention matrix and the two Grad-CAM overlays were reported together for the same test cases to link fusion behavior with spatial evidence in both views.

These visualizations support an interpretable view of both fusion-level interactions and view-specific spatial contributions.

## 4. Results

### 4.1. Model E Results

The proposed DVTF-based classifier with MC-dropout inference and post hoc logistic calibration (Model E) was evaluated using 5-fold stratified group cross-validation at the side level. For each fold, the final checkpoint was selected on the validation subset, and the decision threshold was determined on the validation subset by maximizing the F1-score. Table 1 reports accuracy, precision, recall, F1-score, ROC-AUC, PR-AUC, and negative log-likelihood (NLL).

Because the operating threshold was selected on the validation subset of each fold, the resulting decision threshold is fold-specific $\tau_{f}$ and therefore differs across folds; the per-fold $\tau_{f}$ values are reported in Table S3.

Summary (Model E, mean ± SD over 5 folds)

- Average Accuracy: 96.88% ± 2.39%
- Average F1 Score: 97.68% ± 1.66%
- Average ROC-AUC: 0.9915 ± 0.0098
- Average PR-AUC: 0.9968 ± 0.0029
- Brier = 0.0236 ± 0.0145, ECE = 0.0334 ± 0.0171, NLL = 0.135 ± 0.07

Across folds, the model achieved a mean (±SD) accuracy of 96.88% ± 2.39% and a mean F1-score of 97.68% ± 1.66%. The mean ROC-AUC and PR-AUC were 0.9915 ± 0.0098 and 0.9968 ± 0.0029, respectively, indicating strong separability and consistently high precision–recall behavior. In addition, probability quality was supported by a mean Brier score of 0.0236 ± 0.0145, a mean ECE of 0.0334 ± 0.0171 and a mean NLL of 0.135 ± 0.07, which is consistent with well-behaved confidence estimates under calibration.

**Figure 5:**
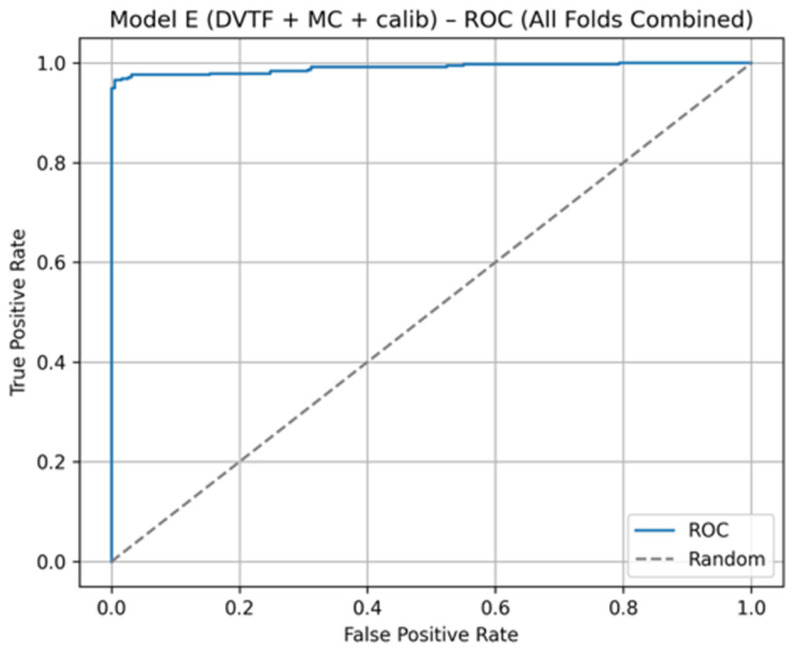
Model E: ROC curve computed by aggregating the five held-out test folds.

**Figure 6:**
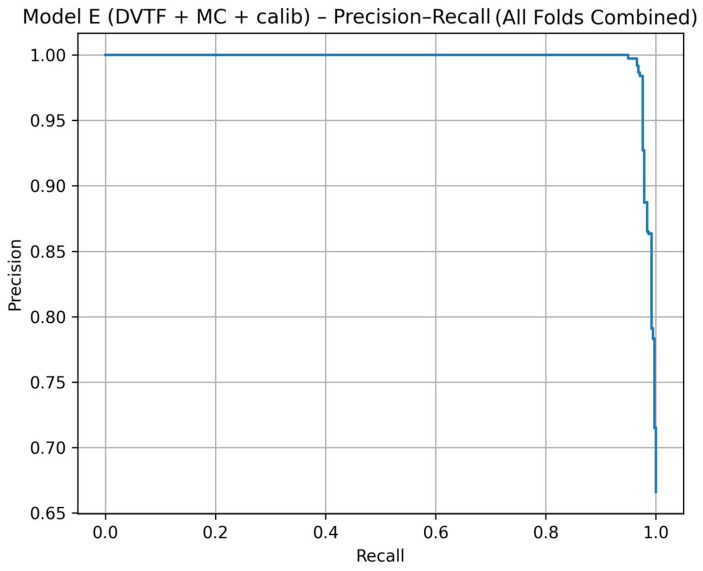
Model E: precision–recall curve computed by aggregating the five held-out test folds.

**Figure 7:**
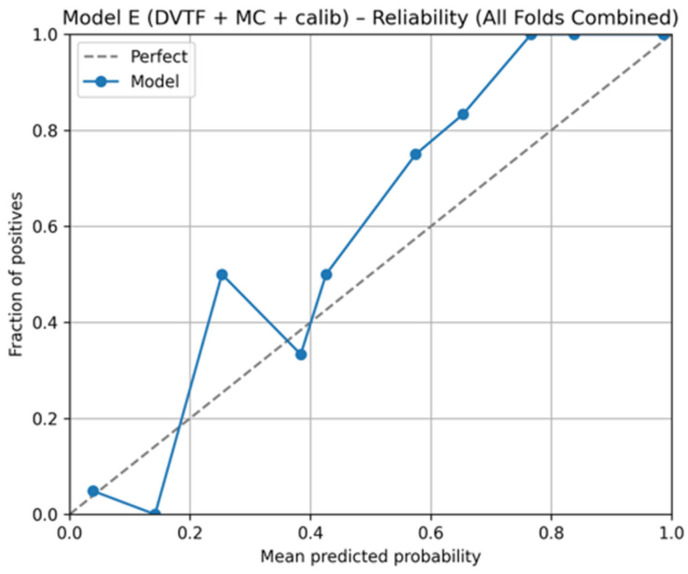
Model E: reliability diagram for calibrated probabilities, aggregated across the five held-out test folds.

**Figure 8:**
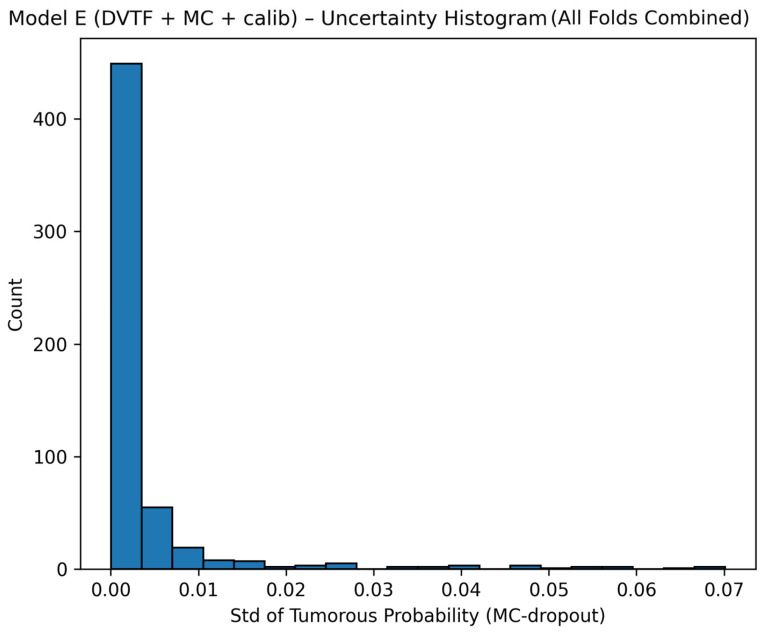
Model E: uncertainty histogram showing the distribution of the standard deviation of the tumorous probability across stochastic forward passes, aggregated across the five held-out test folds.

Fold 1 achieved the highest accuracy (0.9907) and the highest ROC-AUC (1.0000), whereas Fold 5 produced the lowest accuracy (0.9333). Figure 5, Figure 6, Figure 7 and Figure 8 summarize the aggregated ROC curve, precision–recall curve, reliability diagram (after calibration), and MC-dropout uncertainty distribution across the five held-out test folds. This set of results indicates that the proposed configuration achieves high discrimination while maintaining stable probabilistic outputs.

Table 2 summarizes the pooled confusion matrix for Model E across the five held-out folds. The aggregated counts were TN = 183, FP = 6, FN = 12, and TP = 365 (*N* = 566). Thus, misclassifications were limited, with false positives ranging from 0 to 3 per fold and false negatives ranging from 1 to 5 per fold. Per-fold confusion matrices are provided in Supplementary Table S3.

Sensitivity at fixed specificity (0.90, 0.95, and 0.97) was evaluated fold-wise to provide clinically interpretable operating points. For each fold, a decision threshold was selected on the validation subset to achieve at least the target specificity and was then applied to the held-out test subset. Mean ± SD of test specificity, test sensitivity, and the selected validation threshold are reported in Table S4.

To summarize discrimination and probability behavior across cross-validation, test predictions from the five held-out folds were aggregated. The resulting ROC curve is shown in Figure 5, and the corresponding precision–recall curve is shown in Figure 6. Calibration behavior after post hoc logistic mapping is shown in Figure 7 using a reliability diagram. Uncertainty estimates obtained from MC-dropout are summarized in Figure 8 as the distribution of the standard deviation of the tumorous probability across stochastic forward passes. These plots support the fold-wise performance reported in Table 1.

### 4.2. Ablation Study Results (Models A–E)

Table 3 summarizes the mean ± SD performance across five folds for Models A–E and the additional fusion baselines. Relative to the single-view baseline (Model A), Model B improved all key metrics, indicating that incorporating paired CC/MLO information contributes to classification performance. Model C achieved the highest mean ROC-AUC among the deterministic variants, which suggests that token-level interaction improves separability beyond simple feature concatenation. Model D introduced uncertainty estimation via MC-dropout, and Model E further applied logistic calibration. In this setting, Model E preserved strong discrimination while achieving the lowest Brier score and the lowest ECE overall, indicating improved probabilistic accuracy after calibration.

For the additional fusion baselines, gated fusion achieved discrimination close to Model E but showed higher calibration error. Weighted token fusion reduced both discrimination and calibration quality. Cross-attention achieved the lowest mean ECE among the fusion baselines; however, its specificity was lower and substantially more variable, which reduced overall accuracy. Overall, the DVTF-based design (Model E) provided the most consistent trade-off between discrimination and calibrated probability estimates in this study.

### 4.3. Resolution Sensitivity Analysis

To assess sensitivity to input resolution, Model E was re-trained and evaluated using the same 5-fold stratified group cross-validation protocol while changing the input size from 224 × 224 to 384 × 384. Performance at 384 × 384 remained comparable and slightly improved in discrimination metrics (Acc, F1, ROC-AUC, PR-AUC). Probability quality metrics (Brier, ECE) were of similar magnitude, with small differences across settings. Overall, these results suggest that the reported performance is not dependent on the chosen input resolution under the current preprocessing and training configuration. Table 4 summarizes resolution sensitivity for Model E.

### 4.4. K-Sensitivity Analysis and Inference Time

The sensitivity of Model E to the number of MC-dropout passes (K) was evaluated using an independent rerun of the same pipeline under the same cross-validation protocol. This design was used to measure the stability of discrimination and calibration as K varies, while accepting small absolute differences relative to the main Model E run reported in Table 3.

Discrimination remained stable across K ∈ {5, 10, 20}. ROC-AUC ranged from 0.9845 to 0.9851, while PR-AUC ranged from 0.9938 to 0.9941 (Table 5 and Table S5). Calibration varied modestly across K, with the lowest mean ECE observed at K = 10 (Table 5 and Figure S2). Brier scores were similar across K, with only small differences (Figure S1).

In contrast, inference cost increased approximately linearly with K. Mean latency increased from 21.3 ms/sample at K = 5 to 42.9 ms/sample at K = 10 and 85.0 ms/sample at K = 20 (Table 5 and Table S6). Based on this trade-off, K = 10 was retained as the default setting for subsequent experiments, as it preserves stable calibration while maintaining feasible inference time. Discrimination and calibration trends are summarized in Figures S1–S3.

This K-sensitivity analysis was obtained from an independent rerun of Model E under the same evaluation protocol; therefore, the absolute values at K = 10 may differ slightly from the main Model E results reported in Table 3.

### 4.5. Fusion Token Attention and Grad-CAM Examples (Model E, K = 10)

Representative held-out test cases were visualized to demonstrate both fusion behavior and spatial evidence across views. Each case is reported using (i) the token attention matrix from the final fusion layer and (ii) Grad-CAM overlays for the CC and MLO views from the ResNet stream.

Cases were categorized as TP/FP/FN (and TN when reported) using the fold-specific operating threshold $\tau_{f}$ that was selected on the validation subset by maximizing F1, consistent with the thresholds used for the reported confusion matrices and scalar metrics.

**Figure 9:**
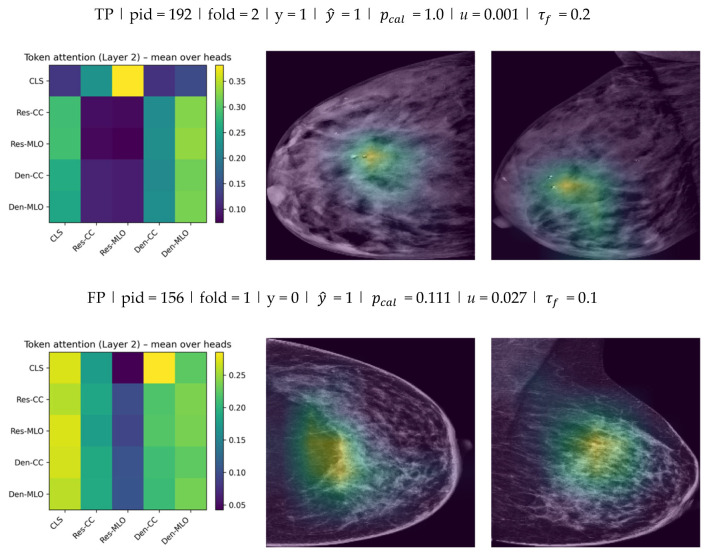

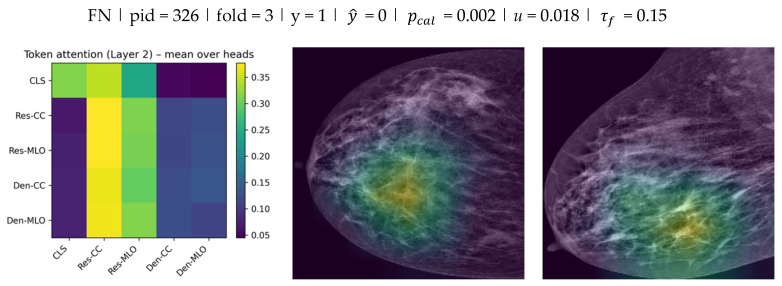
Fusion token attention (Layer 2) and Grad-CAM examples for Model E (K=10) across TP, FP, and FN test cases.

As shown in Figure 9, the fusion attention matrices indicate how the model distributes attention across CC/MLO and backbone-specific tokens, while Grad-CAM highlights the spatial regions that contributed most strongly to the stream-level evidence in each view. Overall, the qualitative results suggest that the model integrates information from both views at the fusion stage and that the spatial evidence is not confined to a single projection.

This combined visualization provides complementary fusion-level and spatial-level interpretability for Model E.

For each case, the left panel reports the token-to-token attention matrix from the final fusion transformer layer (mean over heads) for the five tokens $\left[CLS\right],\;Res-CC,\;Res-MLO,\;Den-CC,\;Den-MLO$ (rows: query; columns: key). The middle and right panels show Grad-CAM overlays on the low-energy CC and MLO images from the ResNet stream. Each example is annotated with y, $\hat{y}$, calibrated probability $p_{cal}$, and uncertainty u (standard deviation across K stochastic passes). Discrete predictions were obtained using the fold-specific validation-selected threshold $\tau_{f}$, which was then applied unchanged to the corresponding test subset.

## 5. Discussion

Recent CESM studies have reported strong discrimination using attention mechanisms, fusion strategies, and combinations of imaging with clinical variables, depending on the task definition and endpoint [20,21].

However, many pipelines still evaluate views with limited cross-view interaction or do not report uncertainty-related outputs. This motivates models that (i) fuse CC and MLO features explicitly and (ii) provide probability outputs that are more informative for downstream decision support.

In this work, DVTF is reported using the calibrated MC-dropout configuration (Model E). The model combines dual-backbone convolutional feature extraction with transformer-based fusion and then estimates uncertainty using stochastic forward passes during inference. Uncertainty quantification methods of this type have been reviewed as practical additions for medical imaging models when probability outputs are required [22].

### 5.1. Comparative Evaluation with Prior CESM-Based Studies

Table 6 summarizes representative CESM-related studies that are commonly cited for deep learning or machine learning analysis, together with the main metrics that were reported in those works. 

The table is only intended as a contextual comparison because datasets, endpoints, and evaluation protocols differ across studies.

In Table 6, Model E shows high mean discrimination while operating on standard dual-view low-energy inputs. The main practical difference is that uncertainty and calibration metrics are also reported in this study, whereas many prior CESM papers focus primarily on accuracy and AUC without probability quality measures.

### 5.2. Performance and Architectural Insights

The ablation results (Table 3) support the contribution of each model component. First, moving from a single-stream baseline to explicit dual-view learning improved side-level discrimination, which is consistent with the clinical role of complementary CC and MLO information. Second, adding transformer-based fusion maintained strong ROC and PR behavior (Figure 5 and Figure 6) while providing a structured mechanism for cross-view interaction. 

Third, MC-dropout enabled uncertainty estimation through repeated stochastic forward passes, and post hoc logistic calibration improved probability quality as reflected by the reduced Brier score and ECE in Model E relative to earlier variants (Table 3). Overall, the ablation indicates that the final configuration improves probability usefulness while maintaining high discrimination.

### 5.3. Uncertainty Estimation and Practical Relevance

Uncertainty was quantified as the standard deviation of the tumorous probability across MC-dropout passes. The combined uncertainty histogram (Figure 8) shows that most predictions concentrate at low uncertainty, with a smaller tail of higher-uncertainty cases. This pattern is expected when the model is confident for many samples but remains uncertain for a minority of difficult cases. 

The reliability diagram (Figure 7) provides a complementary view by comparing mean predicted probabilities with observed outcome frequency after calibration. Deviations from the diagonal indicate remaining miscalibration in specific probability ranges. These outputs can support a simple workflow where high-uncertainty or poorly calibrated probability regions are prioritized for secondary review, rather than being treated as equally reliable as low-uncertainty predictions.

### 5.4. Limitations

Several limitations should be considered. First, the dataset size (326 patients) and retrospective design may limit cross-site generalization.

Second, Model E was evaluated using low-energy images only. Cross-energy or recombined-image fusion may provide additional contrast information and has been explored in prior work on cross-energy feature fusion [26].

Third, the cross-paper results summarized in Table 6 are included for context only and cannot be treated as a direct benchmark because task definitions, datasets, and evaluation protocols vary across studies.

In addition, clinical factors such as acquisition protocol and contrast medium usage can influence lesion conspicuity and should be considered when comparing results across sites [27].

No explicit domain-shift analysis across scanners or centers was performed; thus, generalization under different acquisition settings remains unverified. From a practical perspective, MC-dropout introduces an approximately linear runtime overhead with K (Table S6), and this trade-off should be considered for clinical deployment.

Moreover, prior CESM studies differ in the image representations used (e.g., low-energy only versus recombined representations), which can affect reported performance and limits strict comparability across publications [15].

Furthermore, this study did not include a reader study or prospective workflow evaluation. Future work may include external validation, decision-threshold evaluation under different operating points, and explicit explainability visualizations to support clinical review.

Finally, as a consequence of the endpoint definition, the reported performance should be interpreted as Normal vs. Tumorous triage performance rather than malignant-only classification performance.

### 5.5. Summary

This study reported a dual-view fusion framework for side-level classification using low-energy (DM) images from CESM acquisitions and evaluated it using patient-grouped cross-validation. The final model configuration (Model E) combined dual-backbone feature extraction, transformer fusion, MC-dropout uncertainty, and logistic calibration. The resulting performance remained high while also reporting probability quality and uncertainty outputs, which are needed when probabilities are intended for decision support.

## 6. Conclusions

This study presented a dual-view breast-side classification approach using low-energy (DM) images from CESM acquisitions that fuses CC and MLO representations using a transformer-based module and evaluates performance at the breast-side level under patient-grouped 5-fold cross-validation. The final model configuration combined dual-backbone feature extraction, transformer fusion, MC-dropout inference, and post hoc logistic calibration. Across folds, the model achieved high discrimination and stable precision–recall behavior, while also reporting probability quality using Brier score and ECE. The ablation results indicated that explicit dual-view learning and transformer fusion contributed to performance, and that uncertainty and calibration components primarily improved probability reliability rather than changing discrimination. Several limitations remain, including the retrospective single-center design, evaluation on low-energy inputs only, and the absence of external validation and reader studies. Future work should therefore focus on multi-center validation, operating-point analysis under clinical constraints, and workflow-oriented evaluation that assesses how calibrated probabilities and uncertainty estimates can support secondary review.

## Figures and Tables

**Table 1:** Cross-Validation Performance Metrics.

Fold	Accuracy	Precision	Recall	F1 Score	ROC-AUC	PR-AUC	NLL
1	0.9907	1.0000	0.9861	0.9930	1.0000	1.0000	0.1170
2	0.9820	0.9857	0.9857	0.9857	0.9969	0.9984	0.1096
3	0.9826	1.0000	0.9733	0.9865	0.9953	0.9977	0.0483
4	0.9554	0.9722	0.9589	0.9655	0.9902	0.9955	0.1613
5	0.9333	0.9647	0.9425	0.9535	0.9753	0.9924	0.2383

**Table 2:** Pooled confusion matrix for Model E (MC-dropout + calibration) computed from aggregated out-of-fold test predictions across five held-out folds (Normal = negative class; Tumorous = positive class).

True\Predicted	Normal	Tumorous	Total
Normal	183	6	189
Tumorous	12	365	377
Total	195	371	566

**Table 3:** Ablation and fusion baseline results (mean ± SD across five folds). Models A–E and the additional fusion baselines were evaluated under the same patient-disjoint outer cross-validation protocol. For uncertainty-aware rows (Model D, Model E, and the fusion baselines), results are reported using MC-dropout (K = 10), and calibrated probabilities are reported when logistic calibration is applied.

Model	Acc (%)	Sen (%)	Spe (%)	F1 (%)	ROC-AUC	PR-AUC	Brier	ECE
Model A	89.10 ± 3.21	92.60 ± 4.60	81.80 ± 5.41	91.70 ± 2.70	0.9540 ± 0.0232	0.9772 ± 0.0134	0.0965 ± 0.0419	0.0960 ± 0.0482
Model B	95.95 ± 1.69	95.98 ± 2.14	95.30 ± 1.59	97.00 ± 1.17	0.9830 ± 0.0101	0.9930 ± 0.0027	0.0455 ± 0.0208	0.0490 ± 0.0216
Model C	94.70 ± 1.41	94.10 ± 2.67	95.40 ± 1.45	96.00 ± 1.11	0.9880 ± 0.0057	0.9948 ± 0.0021	0.0648 ± 0.0211	0.0710 ± 0.0278
Model D	94.30 ± 1.87	96.05 ± 2.65	90.70 ± 3.09	95.80 ± 1.34	0.9867 ± 0.0077	0.9943 ± 0.0026	0.0468 ± 0.0168	0.0515 ± 0.0156
Model E	96.88 ± 2.39	96.93 ± 1.87	96.67 ± 3.85	97.68 ± 1.66	0.9915 ± 0.0098	0.9968 ± 0.0029	0.0236 ± 0.0145	0.0334 ± 0.0171
Weighted token	93.85 ± 2.76	93.90 ± 4.09	92.90 ± 4.48	95.30 ± 2.15	0.9790 ± 0.0125	0.9913 ± 0.0049	0.0555 ± 0.0396	0.0810 ± 0.0795
Gated	95.00 ± 1.03	95.55 ± 1.76	93.20 ± 1.82	96.15 ± 0.81	0.9863 ± 0.0037	0.9938 ± 0.0010	0.0420 ± 0.0152	0.0610 ± 0.0448
Cross-attention	92.00 ± 4.39	95.00 ± 1.45	86.30 ± 13.32	94.05 ± 3.13	0.9826 ± 0.0068	0.9926 ± 0.0025	0.0462 ± 0.0108	0.0415 ± 0.0078

**Table 4:** Resolution sensitivity for Model E (mean ± SD over 5 folds).

Setting	Acc (%)	F1 (%)	ROC-AUC	PR-AUC	Brier	ECE
224 × 224 (main)	96.88 ± 2.39	97.68 ± 1.66	0.9915 ± 0.0098	0.9968 ± 0.0029	0.0236 ± 0.0145	0.0334 ± 0.0171
384 × 384 (sensitivity)	97.34 ± 0.97	98.01 ± 0.70	0.9937 ± 0.0067	0.9976 ± 0.0020	0.0252 ± 0.0081	0.0357 ± 0.0069

**Table 5:** Sensitivity of Model E to the number of MC-dropout passes (K). Metrics are reported as mean ± standard deviation across five stratified, patient-disjoint folds. Latency is measured on an NVIDIA A100 (batch size = 16) and reported per side-level sample.

K	ROC-AUC (Mean ± SD)	PR-AUC (Mean ± SD)	Brier (Mean ± SD)	ECE (Mean ± SD)	Latency (ms/Sample) (Mean ± SD)
5	0.9845 ± 0.0067	0.9939 ± 0.0024	0.0363 ± 0.0148	0.0362 ± 0.0143	21.3 ± 2.9
10	0.9845 ± 0.0075	0.9938 ± 0.0029	0.0373 ± 0.0152	0.0338 ± 0.0130	42.9 ± 5.1
20	0.9851 ± 0.0077	0.9941 ± 0.0028	0.0364 ± 0.0147	0.0345 ± 0.0130	85.0 ± 9.9

**Table 6:** Representative CESM-related studies and reported performance (metrics as reported; NR = not reported).

Study	Approach (Brief)	Inputs	Accuracy (%)	AUC	PR-AUC	Notes
[20]	Attention-based deep learning (multicenter)	CESM (single-view)	91.0	0.970	NR	Task/protocol differs by study
[21]	Deep learning + clinical-pathological nomogram	CESM + clinical	93.8	0.982	0.983	Clinical variable fusion
[23]	Fusion of real and synthetic subtracted CEM	Subtracted CEM	94.1	0.985	0.984	Synthetic augmentation used
[24]	ML/DL on radiomics from CEM and DCE-MRI	CEM + MRI	93.3	0.979	NR	Multimodal acquisition
[25]	Interpretable ML for CESM-based prediction	CESM	94.7	0.987	0.988	Interpretable model emphasis
Proposed (Model E)	Dual-backbone + transformer fusion + MC-dropout + logistic calibration	LE CC + MLO (dual-view)	96.88 ± 2.39	0.9915 ± 0.0098	0.9968 ± 0.0029	Uncertainty + calibrated probabilities

## Data Availability

The data analyzed in this study are available from the Categorized Digital Database for Low Energy and Subtracted Contrast-Enhanced Spectral Mammography (CDD-CESM) hosted on The Cancer Imaging Archive (TCIA), at ref. [10] and the TCIA collection record for access details.

## Supplementary Materials

The following supporting information can be downloaded at https://www.mdpi.com/article/10.3390/jimaging12010041/s1. The supporting information provided with this manuscript: Table S1: CDD-CESM composition and derived low-energy study sample (after side-level worst-case label aggregation across available CC/MLO views); Table S2: Summary of experimental settings and model definitions for Models A–E and the additional fusion base-lines; Table S3: Per-fold confusion matrices (Model E, MC-dropout + calibration); Table S4: Sensitivity at fixed specificity operating points for Model E (MC-dropout + calibration) across five folds; Table S5: Full performance, calibration, and latency metrics for the K-sensitivity analysis of Model E (DVTF + MC-dropout + logistic calibration); Table S6: Inference latency of Model E versus the number of MC-dropout passes (K); Figure S1: Brier score versus the number of MC-dropout passes (K); Figure S2: Expected Calibration Error (ECE) versus the number of MC-dropout passes (K); Figure S3: Discrimination trends versus K (ROC-AUC and/or PR-AUC), reported across five folds (mean ± standard deviation).

## Abbreviations

The following abbreviations are used in this manuscript:

AUC	Area Under the Curve
CC	Craniocaudal
CDD-CESM	Categorized Digital Database for Low Energy and Subtracted Contrast-Enhanced Spectral Mammography
CEM	Contrast-Enhanced Mammography
CESM	Contrast-Enhanced Spectral Mammography
CLAHE	Contrast-Limited Adaptive Histogram Equalization
CLAIM	Checklist for Artificial Intelligence in Medical Imaging
CNN	Convolutional Neural Network
DCE-MRI	Dynamic Contrast-Enhanced Magnetic Resonance Imaging
DL	Deep Learning
DVTF	Dual-View Transformer Fusion
ECE	Expected Calibration Error
FFDM	Full-Field Digital Mammography
FN	False Negative
FP	False Positive
MC	Monte Carlo
MC Dropout	Monte Carlo Dropout
ML	Machine Learning
MLO	Mediolateral Oblique
MRI	Magnetic Resonance Imaging
NLL	Negative Log-Likelihood
PR	Precision–Recall
PR-AUC	Area Under the Precision–Recall Curve
ROC	Receiver Operating Characteristic
ROC-AUC	Area Under the Receiver Operating Characteristic Curve
ROI	Region of Interest
TCIA	The Cancer Imaging Archive
TN	True Negative
TP	True Positive
TRIPOD + AI	Transparent Reporting of a multivariable prediction model for Individual Prognosis Or Diagnosis + Artificial Intelligence (extension)
ViT	Vision Transformer
