# Supplementary material for: Calibrated Transformer Fusion for Dual-View Low-Energy CESM Classification
Source: J Imaging. 2026 Jan 13;12(1):41. doi: 10.3390/jimaging12010041 (PMC12842785; doi:10.3390/jimaging12010041)
Supplement: Supplementary file 1 [file jimaging-12-00041-s001.zip › jimaging-4053590-supplementary.pdf]

Journal of Imaging

# Supplementary Materials

**Supplementary Materials:** Table S1: CDD-CESM composition and derived low-energy study sample (after side-level worst-case label aggregation across available CC/MLO views); Table S2: Summary of experimental settings and model definitions for Models A–E and the additional fusion baselines; Table S3: Per-fold confusion matrices (Model E, MC-dropout + calibration); Table S4: Sensitivity at fixed specificity operating points for Model E (MC-dropout + calibration) across five folds; Table S5: Full performance, calibration, and latency metrics for the K-sensitivity analysis of Model E (DVTF + MC-dropout + logistic calibration); Table S6: Inference latency of Model E versus the number of MC-dropout passes (K); Figure S1: Brier score versus the number of MC-dropout passes (K); Figure S2: Expected Calibration Error (ECE) versus the number of MC-dropout passes (K); Figure S3: Discrimination trends versus K (ROC-AUC and/or PR-AUC), reported across five folds (mean ± standard deviation).

**Table S1.** CDD-CESM composition and derived low-energy study sample (after side-level worst-case label aggregation across available CC/MLO views)

| Category | Item | Value |
|---|---|---|
| Raw dataset | Patients | 326 |
| Raw dataset | Total images | 2,006 |
| Raw dataset | Low-energy (DM) images | 1,003 |
| Raw dataset | CESM images | 1,003 |
| Raw dataset | CC views (all images) | 1,000 |
| Raw dataset | MLO views (all images) | 1,006 |
| Raw dataset | Left / Right images | 1,004 / 1,002 |
| Acquisition | Machine 1 / Machine 2 (patients) | 308 / 18 |
| DM side-level | Breast-side samples (DM) | 566 |
| DM side-level | Complete CC+MLO sides | 435 |
| DM side-level | Single-view sides (total) | 131 |
| DM side-level | CC-only / MLO-only sides | 65 / 66 |
| Labels (DM, side-level) | Normal / Benign / Malignant | 189 / 194 / 183 |
| Labels (DM, binary) | Normal / Tumorous | 189 / 377 |

**Table S2.** Summary of experimental settings and model definitions for Models A–E and the additional fusion baselines; MC-dropout (K=10) and logistic calibration are applied as specified in the table.

| Category | Parameter | Value |
|---|---|---|
| Environment | Platform | Google Colab |
| Environment | GPU | NVIDIA A100-SXM4-40GB (40 GB) |
| Environment | Python | 3.12.12 |
| Environment | PyTorch | 2.9.0+cu126 |
| Environment | CUDA (PyTorch) | 12.6 |
| Environment | cuDNN | 91002 |
| Environment | Seed | 42 |
| CV protocol | Scheme | StratifiedGroupKFold (group = patient ID) |
| CV protocol | Folds | 5 |
| Within-fold split | Validation fraction | 15% of patients in training fold, created using a stratified group split (patient ID) |
| Data / Input | Tensor source | Pre-extracted per-side tensors (*.pt) loaded from Google Drive |

| | | |
|---|---|---|
| Data / Input | Tensor shape assumption | data["tensor"] has shape (C, H, W) with C ≥ 2 |
| Data / Input | Channels used | ch0 = CC LE, ch1 = MLO LE |
| Data / Input | Input size | 224×224 (main); 384×384 (resolution sensitivity, run as a separate experiment) |
| Data / Input | View conversion | Grayscale → pseudo-RGB by channel replication (applied at load time) |
| Data / Input | Input normalization | ImageNet mean/std normalization (applied at load time) |
| Labels / task | Task | Side-level binary classification (Normal vs Tumorous) |
| Labels / task | Label mapping | Normal = 0; Tumorous = 1 (Benign + Malignant merged) |
| Labels / task | Patient ID | data["patient_id"] if present; else filename prefix before first underscore |
| Data loading | Batch size | 16 |
| Data loading | Train sampler | WeightedRandomSampler (inverse-frequency; replacement=True) |
| Augmentation | Flip | Horizontal, p=0.5 |
| Augmentation | Rotation | ±10° |
| Training budget | Max epochs | 60 |
| Early stopping | Metric | Validation ROC-AUC |
| Early stopping | Patience | 10 |
| Optimizer | Type | Adam |
| Optimizer | Learning rate | 1e-4 |
| Optimizer | Weight decay | 1e-4 |
| Scheduler | Type | CosineAnnealingLR |
| Scheduler | T_max / η_min | 30 / 1e-6 |
| Loss | Criterion | Class-weighted CrossEntropyLoss (inverse-frequency weights from training fold) |
| Thresholding | Rule | Maximize validation F1 |
| Thresholding | Sweep | 0.05–0.95 step 0.05 |
| ECE | Bins | 10 (equal-width) |
| Metrics | Reported | ACC, SEN, SPE, PREC, F1, ROC-AUC, PR-AUC, Brier, ECE, NLL |
| Uncertainty | Definition | std of Tumorous probability across K passes (Models D/E and fusion baselines) |
| Uncertainty | Analysis | K ∈ {5,10,20} (Model E K-sensitivity study); fusion baselines evaluated at K=10 |
| MC-dropout | Passes (K) | 10 (Models D/E and fusion baselines) |
| MC-dropout | How enabled | Model set to eval; all nn.Dropout modules switched to train mode (Models D/E and fusion baselines) |
| MC-dropout | Dropout p | 0.2 in DVTF blocks (Models C/D/E); 0.3 in DenseNet heads (Models A/B); 0.2 in fusion-baseline heads/blocks |
| Calibration | Method | Logistic calibration on MC mean Tumorous probabilities (per fold, validation-based) (Model E and fusion baselines) |
| Calibration | Mapping | logit($p$) = a·logit(p_mc) + b (Model E and fusion baselines) |
| Calibration | Fit objective | BCE loss on validation set (Model E and fusion baselines) |
| Calibration | Optimizer / steps | Adam, lr=0.01, 500 steps (Model E and fusion baselines) |
| Calibration | Applied to | Fold test set MC-mean probabilities (Model E and fusion baselines) |
| Feature extractor (DVTF & baselines) | ResNet-101 embedding | ResNet-101 conv trunk → GAP (2048) → Linear 2048→1024 → L2-norm (Models C/D/E and fusion baselines) |
| Feature extractor (DVTF & baselines) | DenseNet-121 embedding | DenseNet-121 features → GAP (1024) → Linear 1024→1024 → L2-norm (Models C/D/E and fusion baselines) |
| Transformer fusion (DVTF) | Tokens per sample | 4 image tokens (CC/MLO × ResNet/DenseNet) + 1 learnable [CLS] = 5 tokens (Models C/D/E) |

| | | |
|---|---|---|
| Transformer fusion | Positional embedding | Learnable positional encoding for 5 tokens (Models C/D/E) |
| Transformer fusion | Transformer encoder layers | 2 (Models C/D/E) |
| Transformer fusion | Attention heads (h) | 4 (Models C/D/E) |
| Transformer fusion | Model dimension (d) | 1024 (Models C/D/E) |
| Transformer fusion | Feed-forward dimension | 4096 (Models C/D/E) |
| Transformer fusion | Activation | GELU (Models C/D/E) |
| Transformer fusion | Dropout | 0.2 (Models C/D/E) |
| Model A | Definition | Single-view CNN baseline (CC LE only), deterministic |
| Model A | Views used | CC only |
| Model A | Tensor channels | ch0 only |
| Model A | Backbone | DenseNet-121 (ImageNet pretrained) |
| Model A | Fusion | None (single stream) |
| Model A | Feature size | 1024 (DenseNet pooled) |
| Model A | Head | 1024→256→2, ReLU, Dropout 0.3 |
| Model A | Attention/Transformer | No |
| Model A | Uncertainty | None (deterministic softmax) |
| Model A | Calibration | None |
| Model B | Definition | Dual-view CNN baseline (CC+MLO LE), deterministic |
| Model B | Views used | CC + MLO |
| Model B | Tensor channels | ch0 (CC) + ch1 (MLO) |
| Model B | Backbone | DenseNet-121 shared weights for CC and MLO |
| Model B | Fusion | Concatenation of pooled CC and pooled MLO features |
| Model B | Feature size per view | 1024 |
| Model B | Fused feature size | 2048 |
| Model B | Head | 2048→256→2, ReLU, Dropout 0.3 |
| Model B | Attention/Transformer | No |
| Model B | Uncertainty | None (deterministic softmax) |
| Model B | Calibration | None |
| Model C | Definition | DVTF-Net (dual-view transformer fusion), no MC-dropout (deterministic test) |
| Model C | Views used | CC + MLO |
| Model C | Tokens | 4 image tokens + [CLS] (5 total) |
| Model C | Backbones | ResNet-101 + DenseNet-121 (ImageNet pretrained) |
| Model C | Fusion | 2-layer Transformer encoder over 5 tokens |
| Model C | Head | [CLS] 1024→256→2, GELU, Dropout 0.2 |
| Model C | Uncertainty | None (deterministic softmax) |
| Model C | Calibration | None |
| Model D | Definition | DVTF-Net + MC-dropout (mean probability + uncertainty) |
| Model D | Inference probability | MC mean Tumorous probability across K=10 passes |
| Model D | Uncertainty | std of Tumorous probability across K=10 passes |
| Model D | Threshold selection | Best F1 on validation MC-mean probabilities |
| Model D | Metrics computed on | Test MC-mean probabilities |
| Model D | Calibration | None |
| Model E | Definition | DVTF-Net + MC-dropout + logistic calibration (per-fold) |

| | | |
|---|---|---|
| Model E | Inference probability | MC mean Tumorous probability → logistic-calibrated Tumorous probability |
| Model E | Calibration fit set | Validation MC-mean probabilities (same fold) |
| Model E | Calibration params saved | (a, b) stored per fold in metrics JSON |
| Model E | Threshold selection | Best F1 on calibrated validation probabilities |
| Model E | Metrics computed on | Test calibrated probabilities |
| Model E | Uncertainty | std of Tumorous probability across K=10 passes (same as Model D) |
| Model E | Parameters | 78.1M |
| Fusion baseline (Weighted) | Definition | Weighted token fusion over four global tokens, with MC-dropout + logistic calibration |
| Fusion baseline (Weighted) | Views used | CC + MLO |
| Fusion baseline (Weighted) | Tokens | 4 global tokens: ResNet(CC), ResNet(MLO), DenseNet(CC), DenseNet(MLO) |
| Fusion baseline (Weighted) | Backbones | ResNet-101 + DenseNet-121 (ImageNet pretrained) |
| Fusion baseline (Weighted) | Fusion | Learnable global weights (softmax over 4 tokens) → weighted sum (1024-D) |
| Fusion baseline (Weighted) | Post-fusion MLP | Dropout 0.2 → Linear 1024→1024 → GELU → Dropout 0.2 |
| Fusion baseline (Weighted) | Head | 1024→256→2, GELU, Dropout 0.2 |
| Fusion baseline (Weighted) | Uncertainty | std of Tumorous probability across K=10 passes |
| Fusion baseline (Weighted) | Calibration | Logistic calibration on MC mean probabilities (per fold, validation-based) |
| Fusion baseline (Gated) | Definition | View-wise feature gating per backbone, with MC-dropout + logistic calibration |
| Fusion baseline (Gated) | Views used | CC + MLO |
| Fusion baseline (Gated) | Backbones | ResNet-101 + DenseNet-121 (ImageNet pretrained) |
| Fusion baseline (Gated) | Gating (per backbone) | g = sigmoid(MLP([CC, MLO])) with g ∈ R^1024; fused = g·CC + (1−g)·MLO |
| Fusion baseline (Gated) | Backbone fusion | Concat [ResNet_fused, DenseNet_fused] (2048-D) → Linear 2048→1024 → GELU → Dropout 0.2 |
| Fusion baseline (Gated) | Head | 1024→256→2, GELU, Dropout 0.2 |
| Fusion baseline (Gated) | Uncertainty | std of Tumorous probability across K=10 passes |
| Fusion baseline (Gated) | Calibration | Logistic calibration on MC mean probabilities (per fold, validation-based) |
| Fusion baseline (Cross-attention) | Definition | Two-token attention between CC and MLO per backbone, with MC-dropout + logistic calibration |
| Fusion baseline (Cross-attention) | Views used | CC + MLO |
| Fusion baseline (Cross-attention) | Tokens (per backbone) | 2 tokens: [CC, MLO] embeddings (1024-D each) |
| Fusion baseline (Cross-attention) | Attention module | Multi-head attention over sequence length 2 (heads=4), with Dropout 0.2 after attention and within FFN |
| Fusion baseline (Cross-attention) | Pooling | Mean pooling over the 2 attended tokens → 1024-D fused vector (per backbone) |
| Fusion baseline (Cross-attention) | Backbone fusion | Concat [ResNet_fused, DenseNet_fused] (2048-D) → Linear 2048→1024 → GELU → Dropout 0.2 |

| | | |
|---|---|---|
| Fusion baseline (Cross-attention) | Head | 1024→256→2, GELU, Dropout 0.2 |
| Fusion baseline (Cross-attention) | Uncertainty | std of Tumorous probability across K=10 passes |
| Fusion baseline (Cross-attention) | Calibration | Logistic calibration on MC mean probabilities (per fold, validation-based) |

**Table S3.** Per-fold confusion matrices (Model E, MC+cal)

| Fold | N | TN | FP | FN | TP | $\tau_f$ |
|---|---|---|---|---|---|---|
| 1 | 108 | 36 | 0 | 1 | 71 | 0.10 |
| 2 | 111 | 40 | 1 | 1 | 69 | 0.20 |
| 3 | 115 | 40 | 0 | 2 | 73 | 0.15 |
| 4 | 112 | 37 | 2 | 3 | 70 | 0.15 |
| 5 | 120 | 30 | 3 | 5 | 82 | 0.15 |

(Positive class = Tumorous; matrix layout = rows true {Normal, Tumorous} × cols predicted {Normal, Tumorous}. Threshold is the fold operating threshold $\tau_f$ used for the reported discrete metrics.)

**Table S4.** Sensitivity at fixed specificity operating points for Model E (MC+cal) across five folds. For each fold, a decision threshold was selected on the validation subset to achieve the target specificity, then applied to the held-out test subset to report test specificity and test sensitivity. Values are reported as mean ± standard deviation across folds. Positive class is Tumorous.

| **Specificity target** | **Test specificity (mean ± SD)** | **Test sensitivity (mean ± SD)** | **Threshold (validation, mean ± SD)** |
|---|---|---|---|
| 0.90 | 0.974 ± 0.031 | 0.951 ± 0.018 | 0.353 ± 0.310 |
| 0.95 | 0.990 ± 0.014 | 0.936 ± 0.033 | 0.559 ± 0.303 |
| 0.97 | 0.990 ± 0.014 | 0.936 ± 0.033 | 0.559 ± 0.303 |

Due to discrete thresholding and the fold-wise score distribution, the ≥0.95 and ≥0.97 constraints resulted in the same selected operating point in this dataset.

**Table S5.** Full performance, calibration, and latency metrics for the K-sensitivity analysis of Model E (DVTF + MC-dropout + logistic calibration). Values are mean ± standard deviation across five patient-disjoint folds.

| K | ACC (%) | SEN (%) | SPE (%) | PREC (%) | F1 (%) | ROC-AUC | PR-AUC | Brier | ECE |
|---|---|---|---|---|---|---|---|---|---|
| 5 | 95.56 ± 1.85 | 95.56 ± 2.04 | 95.78 ± 4.11 | 97.81 ± 2.27 | 96.64 ± 1.38 | 0.9845 ± 0.0067 | 0.9939 ± 0.0024 | 0.0363 ± 0.0148 | 0.0362 ± 0.0143 |
| 10 | 95.76 ± 2.08 | 94.78 ± 2.37 | 97.80 ± 2.08 | 98.88 ± 1.09 | 96.78 ± 1.53 | 0.9845 ± 0.0075 | 0.9938 ± 0.0029 | 0.0373 ± 0.0152 | 0.0338 ± 0.0130 |
| 20 | 95.75 ± 1.34 | 95.01 ± 2.25 | 97.37 ± 2.68 | 98.63 ± 1.52 | 96.76 ± 1.00 | 0.9851 ± 0.0077 | 0.9941 ± 0.0028 | 0.0364 ± 0.0147 | 0.0345 ± 0.0130 |

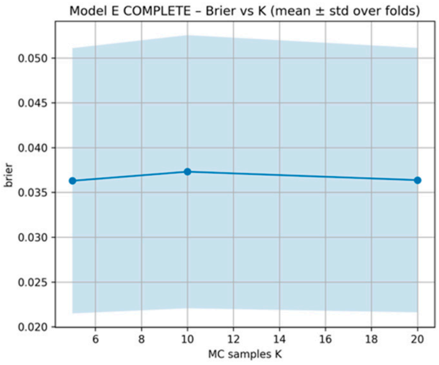


**Figure S1.** Brier score versus the number of MC-dropout passes ($K$).

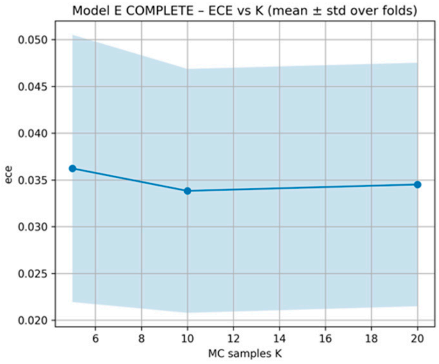


**Figure S2.** Expected Calibration Error (ECE) versus the number of MC-dropout passes ($K$).

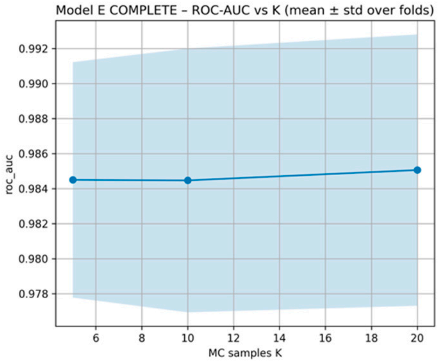


**Figure S3.** Discrimination trends versus $K$ (ROC-AUC and/or PR-AUC), reported across five folds (mean ± standard deviation).

**Table S6.** Inference latency of Model E versus the number of MC-dropout passes (K), measured on an NVIDIA A100 with batch size = 16.

| K | ms/sample (mean ± std) | ms/batch (mean ± std) | samples/s (mean ± std) |
|---|---|---|---|
| 5 | 21.3 ± 2.9 | 298.9 ± 5.4 | 47.0 ± 0.7 |
| 10 | 42.9 ± 5.1 | 578.6 ± 4.7 | 23.3 ± 0.1 |
| 20 | 85.0 ± 9.9 | 1134.2 ± 7.8 | 11.8 ± 0.1 |
